# Implementing a telehealth prehabilitation education session for patients preparing for major cancer surgery

**DOI:** 10.1186/s12913-021-06437-w

**Published:** 2021-05-10

**Authors:** Jamie L. Waterland, Rani Chahal, Hilmy Ismail, Catherine Sinton, Bernhard Riedel, Jill J. Francis, Linda Denehy

**Affiliations:** 1 Department of Anaesthesia, Perioperative and Pain Medicine, Peter MacCallum Cancer Centre, Melbourne, Australia; 2 Department of Physiotherapy, The University of Melbourne, Melbourne, Australia; 3 Division of Allied Health, Peter MacCallum Cancer Centre, Melbourne, Australia; 4 Centre for Integrated Critical Care, The University of Melbourne, Melbourne, Australia; 5 Division of Surgical Oncology, Peter MacCallum Cancer Centre, Melbourne, Australia; 6 School of Health Sciences, The University of Melbourne, Melbourne, Australia; 7 Department of Health Services Research, Peter MacCallum Cancer Centre, Melbourne, Australia

**Keywords:** Prehabilitation, Telehealth, Cancer, Perioperative, Education, Physiotherapy, RE-AIM, Impact

## Abstract

**Background:**

Prehabilitation services assist patients in preparing for surgery, yet access to these services are often limited by geographical factors. Enabling rural and regional patients to access specialist surgical prehabilitation support with the use of telehealth technology has the potential to overcome health inequities and improve post-operative outcomes.

**Aim:**

To evaluate the current and likely future impact of a telehealth preoperative education package for patients preparing for major abdominal cancer surgery.

**Methods:**

A telehealth alternative to a hospital based pre-operative education session was developed and implemented at a dedicated cancer hospital. Adult patients (≥18 years) scheduled for elective major cancer surgery were offered this telehealth alternative. Impact evaluation was conducted using the RE-AIM framework.

**Results:**

To date, 35 participants have consented to participate in the study. Thirty-one participants attended the intervention; 24 (69%) residing in rural or regional areas. Twenty-four (77%) reported that if given a choice they would prefer the online session as opposed to attending the hospital in person. The majority (97%) reported they would recommend the intervention to others preparing for surgery. Session information was recalled by all 26 participants and 77% of participants reported acting on recommendations 2 weeks after the session. Lessons learnt and recommendations for providers implementing similar programs are reported.

**Conclusion:**

Telehealth alternatives to hospital based pre-operative education are well received by patients preparing for major cancer surgery. We make seven recommendations to improve implementation. Further evaluation of implementation strategies alongside clinical effectiveness in future studies is essential.

**Trial registration:**

ACTRN12620000096954, 04/02/2020.

**Supplementary Information:**

The online version contains supplementary material available at 10.1186/s12913-021-06437-w.

## Introduction

Australians diagnosed with cancer who live outside major metropolitan areas are at risk of significantly worse survival outcomes than those in urban areas [1, 2]. Rural and regional patients are less likely to have their treatment overseen by an oncologist [3] and less likely to have access to specialist oncology multidisciplinary services [3, 4]. Travelling long distances to essential cancer services can negatively affect patients’ quality of life [5, 6] and survival [7]. Telehealth provides an opportunity to correct this health inequity [8] and improve health outcomes [9] by offering an alternative for those experiencing travel difficulties associated with caring or work commitments [10, 11], conflicting clinical appointments or symptoms associated with treatment.

Telehealth interventions can vary in their nature from educational or supportive websites, monitoring through electronic questionnaires, to live online consultations [12]. Patient education sessions using audio-visual or multi-media interventions in patients with cancer have been shown to improve satisfaction and knowledge when delivered to patients preparing for cancer surgery [13]. However evidence investigating telehealth in the perioperative setting for patients with cancer is currently limited to small cohort studies [14–17].

Surgical prehabilitation is any intervention given in the pre-operative period that aims to optimise physiological reserve prior to surgery to improve post-operative outcomes [18, 19]. Previously solely focused on exercise, prehabilitation has expanded to include many aspects of clinical care including perioperative education [20]. Multidisciplinary perioperative 60 min group generalised education sessions held in person in the hospital environment, known as ‘Surgery School’, aim to train patients and relatives in aspects of preparation for surgery and postoperative care including nutrition, breathing, physical activity, oral health, psychological support, general health and family support with the aim, when included as part of an enhanced recovery after surgery program, to reduce post operative pulmonary complications (PPCs) [21]. Similar sessions have been conducted at the tertiary hospital where this study was conducted as standard care since 2017. However approximately 30% of eligible patients did not attend due to issues with distance or travel. To address this inequity the present project was designed to implement an alternative service.

The aim of this paper is to evaluate the current and likely future impact of a telehealth preoperative education package for patients preparing for major abdominal cancer surgery using the RE-AIM framework [22] with the exception of the maintenance dimension which will discussed in a later paper.

## Methods

### Participants/target population

Ethics committee approval was obtained for the project (LNR/57280/PMCC-2019) and it was registered on the Australian and New Zealand Clinical Trials Registry (ACTRN12620000096954) on the 04/02/2020 prior to commencing recruitment. Intended users of the telehealth ‘Virtual Surgery School’(VSS) intervention were adult patients preparing for major abdominal cancer surgery.

Patients were eligible to participate if they were ≥ 18 years of age, awaiting abdominal cancer surgery, anticipated to have a > 1 day length of hospital stay with a surgery duration anticipated to be greater than or equal to 120 min and sufficient English language skills to understand study requirements. Potential participants were identified using multidisciplinary meeting notes, pre-anaesthetic clinic lists, distribution of study flyers and clinician referral. Patients were excluded if they had a documented history of cognitive impairment. It was anticipated that greater than 70% of eligible patients would agree to attend the VSS [23]. Informed consent was obtained by an electronic form prior to participating in the study.

### Setting

**Fig. 1:**
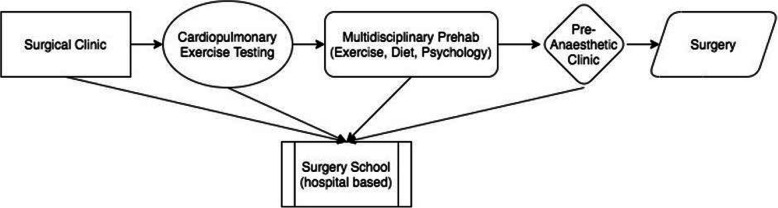
Current Perioperative Prehabilitation Pathway

The VSS intervention was developed by a group of seven clinicians and researchers of the multidisciplinary team within a prehabilitation clinical service at a specialist cancer centre. The team consists of anaesthetists, physiotherapists, dietitians, clinical psychologists, exercise physiologists, specialist nurses and researchers. The team meets weekly and works as a coordinated unit to plan and administer prehabilitation and recovery plans to patients undergoing major cancer surgery (see Fig. 1.).

### Procedure

The project was conducted in four phases. In phase I, the aims were to establish a baseline preoperative pulmonary complication (PPC) prevalence audit and to adapt existing perioperative education materials to a telehealth format. In phase II, the telehealth session was implemented in a pilot phase with consenting patients. Phase II was completed in May 2020, and implementation findings are presented in this paper. In phases III and IV, the intervention will be maintained within a perioperative clinical service with changes based upon the recommendations herein and accompanied by ongoing staff education and monthly audit of PPCs and compliance with post-operative recommendations. Results of phases III and IV will be presented in a later paper.

#### Developing the intervention (phase I)

Development of the intervention followed the below steps:

1. Adapting content from hospital-based education session.
2. Seeking input from consumers regarding intervention content and format.
3. Revising presentations for a telehealth format.
4. Editing content to webinar friendly format.
5. Training presenter to deliver intervention using webinar platform.

Specific aspects of these steps are further described below.

#### Adapting content from the hospital-based session

The hospital-based session is based on previously published iCOUGH [24] and ERAS+ programs [21] and expanded to include additional prehabilitation information with adaptations for the Australian environment. Based on session evaluations, the hospital-based Surgery School (approximately 3 h with breaks) was transformed into a moderated webinar, of 75 min, to promote participant engagement during the length of the session whilst maintaining key content. During adaptation, staff iteratively piloted the webinar format and provided informal feedback on audio, connectivity and ease of use.

#### Seeking consumer input

Consumer participation was sought for refinement of content, however allocation of a consumer representative coincided with the summer holiday period and could not be finalised in time prior to study commencement. Consumer input was based on hospital based session feedback.

#### Training on the webinar platform

All webinar sessions were hosted by one facilitator on the 2020 Zoom Video Communications, Inc.© [25] platform. Training was undertaken by the facilitator on how to deliver the session effectively in the online format. This included familiarisation with webinar content, webinar software training, content rehearsal and simulation of session delivery between members of the research team and staff to ensure functionality and reliability of the webinar platform.

#### Piloting the intervention (phase II)

Eligible participants were offered a choice of the hospital based education session or VSS in preparation for their surgery. Three weeks after the commencement of the intervention, the face-to-face hospital based sessions were no longer possible due to social distancing requirements in response to the COVID-19 pandemic (11th of March 2020). Potential participants were contacted via phone, the study explained and invited to the next VSS via email invitation.

#### The virtual surgery school intervention

The VSS intervention is described below according to the 12 item template for intervention description and replication (TIDieR) checklist [26], with the exception of item 2 which was included in the introduction. TIDieR item descriptions are provided in Additional file 1.

**Fig. 2:**
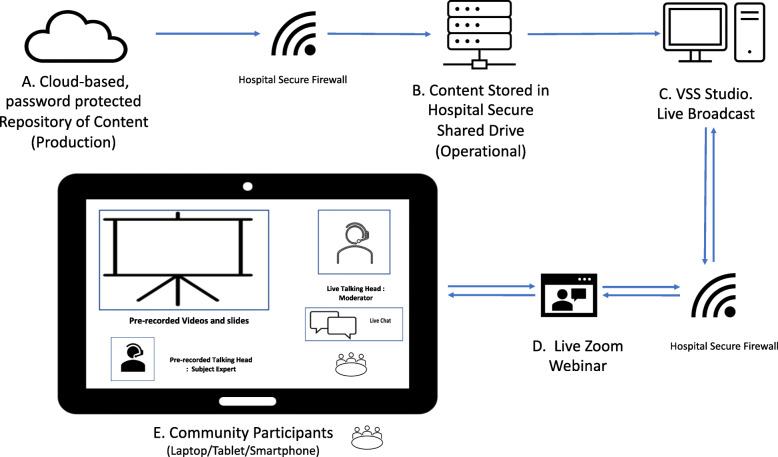
Webinar Technical Set Up

Virtual Surgery School (Item 1) is a single 75 min intervention (Item 8) that was offered bimonthly, with a total of seven sessions delivered. Sessions alternated between morning and afternoon to accommodate participants that may have work or other commitments. Participants were also provided with step-by-step written instructions on how to access the intervention via email in the days leading up to the session. The intervention consisted of pre-recorded slide presentations of targeted prehabilitation topics (see Table 1) that were displayed side-by-side with a video stream of the relevant clinician. A live facilitator, a PhD candidate and physiotherapist with over 3 years of experience in prehabilitation, was included to introduce the sessions and answer questions on the webinar content. The webinar technical set up is given in Fig. 2. The presentations were pre-recorded by senior clinicians in their respective areas of expertise. The introduction presentation was pre-recorded by a consultant anaesthetist and clinical lead of the prehabilitation program with over 15 years’ experience as a consultant anaesthetist. All video presentations presented during the session were made available to participants through an online password-protected video repository hosted on the 2020 Vimeo© website [27] after attending in order to reinforce content and allow for self-education. Instructions to access these videos was were provided via email. (Items 3, 4 and 5).

**Table 1:** VSS Presentations: a brief overview

VSS presentations (in this order);	Key Messages
1. Welcome and evidence for the session	• Introduction to prehabilitation team • Story of previous patient with successful outcome • Encouragement to participate in Surgery School
2. Respiratory care bundle (aCOUGH) including	• Risks of post-operative chest infection: lung collapse, body’s lung cleaning mechanism physiology
Active cycle of breathing technique instructions	• Airway clearance technique including controlled breathing, deep breathing, huff and cough
Oral care	• Attend the dentist/hygienist • Use an alcohol-free antiseptic mouthwash daily • Brushing teeth twice daily
Get out of bed	• Sit up straight in bed and sit out of bed to promote early post-operative mobilisation • Gradual return to activity after surgery
3. Exercise prehabilitation	• Smoking cessation • Benefits of exercise • Recommendations: avoid inactivity, physical activity to meet recommended guidelines, breathing exercises 5–10 min daily. • Monitoring intensity • Early post operative mobilisation. • Tips for safe exercise and starting an exercise program prior to surgery
4. Nutrition before surgery	• Effect of surgery on nutrition • Nutrition to increase muscle stores: exercise + eat/drink protein + eat/drink enough energy • Maintain weight • Follow Healthy Eating Guidelines • Post operative nutrition
5. Pain management after surgery	• Causes of pain after surgery • Daily pain assessment • Commonly used pain relief: PCA, epidural, spinal injections, tablets, wafers, liquids. • Common Side Effects incl: nausea, vomiting, rash/itch
6. Psychological preparedness and relaxation	• Why focus on psychological wellbeing? Prepare for surgery, cope with surgery, recover from surgery • Strategies to manage worry e.g. relaxation strategies • Staying motivated and goal setting • Using family and friend supports

To maximise access to the intervention, VSS was delivered by an online webinar that participants could access anywhere with internet access and an appropriate device, ideally in their own home. Participants in the webinar could view the host and webinar content but for privacy could not see other participants. Additionally only first names were used on the participant list. All participants were encouraged to ask questions through the Q&A function on the webinar or submit them to the facilitator after the session via email. The webinar software was available in computer, tablet and mobile formats. Participants were encouraged to watch the intervention with their support people (Items 6 and 7).

The intervention aimed to give participants a base of knowledge on which future interventions could be individualised and therefore was not tailored to the individual patient (Item 9). The program was not modified during the pilot period; however, findings will be used to modify the ongoing program (Item 10).

Treatment fidelity was assessed using the NIH framework [28] with several strategies employed to improve fidelity. VSS was adapted from the hospital based version, as outlined earlier within the Methods section. This process significantly shortened the duration of the intervention to improve receipt of treatment. Fidelity of delivery was maximised by pre-recording presentations and standardising delivery of content via a checklist. Access to intervention material was provided after the session to improve receipt of treatment. Treatment fidelity outcomes are provided in the results section under the RE-AIM dimension Implementation (Items 11 and 12).

### Measures

#### Patient characteristics

After consent, participants’ demographic data were extracted from their medical record, where available, including gender, date of birth, height, weight, primary language spoken, functional status, proposed surgery and medical history. Burden of comorbidities was calculated using the Charlson Comorbidity Index (CCI) [29], operative risk was calculated using the Johns Hopkins modified Surgical Risk [30] and American Society of Anaesthesiologists (ASA) [31] scores, and risk of PPCs was calculated using the Assess Respiratory Risk in Surgical Patients in Catalonia (ARISCAT) score [32]. All participant data including pre and post session questionnaire responses were collected and stored on the secure REDCap® platform.

#### Pre-session questionnaire

Participants completed the same pre-session questionnaire that is included in the hospital based session. The clinician and researcher developed questionnaire has been in use since 2017 (Additional file 2) and consists of five core and three follow up questions. Participants completed three items on a five point likert scale (ranging from strongly disagree to strongly agree) aimed at assessing patient satisfaction and based on statements previously used to evaluate pre-operative education [33]. Additional questions provided further demographic data including highest level of education and history of smoking.

#### Webinar analytics

Webinar duration, participant attendance, viewing time and any chat or questions were obtained by attendee and Q&A reports through the Zoom platform. All webinars were recorded for study records. Standardised checklists were completed by the facilitator in each session. Participants who did not attend were contacted by email and/or phone and reason for non-attendance recorded. Session presentations were uploaded to a video repository as individual files after the session. Data on number of videos accessed as well as duration of view were recorded by website analytics.

#### Post-session questionnaire

A post-session evaluation questionnaire was developed specifically for this study by the research team (Additional file 2). The questionnaire consisted of 16 questions and was emailed to all participants at the conclusion of the webinar. The questionnaire was split into three sections, the first: repetition of the three satisfaction items included in the pre-session questionnaire. This was followed by an evaluation on a five point scale (from poor to excellent) for each presentation included in the webinar.

The last section of the evaluation questionnaire assessed satisfaction and logistical elements including where participants would have preferred to attend Surgery School (at the hospital, online at home, or online at a community centre) and then Yes/No questions including whether participants would recommend VSS to others preparing for surgery; whether assistance was needed to connect to the webinar; ease of use; and whether participants watched with others. The evaluation questionnaire concluded with a free text box for any additional comments. The aim of this questionnaire was to identify and assess key areas for improvement of the intervention.

#### Retention phone call

Retention of webinar content was assessed by a scripted phone call (Additional file 2), conducted by a senior nurse, 2 weeks after VSS or in the days leading up to surgery in the case of imminent surgery. Interview methodology and questions were based on a recent study investigating pre-operative education retention [34]. Questions were adapted to the intervention specifics and developed to access unprompted recall and then probe for recognition of session elements and recommendations (Additional file 2). Interview content was reviewed by multidisciplinary members of the research team before use. The phone call began by asking participants to report whether they had accessed the videos since the webinar, which videos and how often. To prevent biasing interview responses, interviews were conducted by a member of the research team that did not conduct the webinar. Interviews were audio-recorded and questions categorised with frequency of responses calculated. The aim of the retention phone call was to investigate the proportion of participants who could recall aspects of the intervention (treatment receipt) and the proportion of participants who acted on recommendations provided during the intervention (enactment of treatment skills).

### Analytic approach

Sociodemographic, clinical data, participation rates and categorised interview responses were analysed using descriptive statistics. Wilcoxon-signed ranked tests were used to assess differences in common questions in pre and post session questionnaires. Quantitative data were then integrated and organised along the RE-AIM dimensions to evaluate the program impact and NIH-recommended fidelity domains [28] to report on ways to further enhance intervention fidelity.

Implementation in this paper is defined as the initiation of a health intervention rather than a widespread upscaling and thus this section focused on intervention fidelity. The RE-AIM (reach, efficacy, adoption, implementation and maintenance) framework was chosen as it has been validated as a tool to plan, evaluate and to assess the impact of a variety of health care and health prevention programs [35–37]. Briefly, ‘reach’ refers to participation and representativeness of the target population. ‘Efficacy’ or effectiveness refers to whether the program influenced the targeted outcomes. ‘Adoption’ refers to the uptake by agencies and settings. ‘Implementation’ (which focuses on fidelity) refers to the extent to which the intervention was delivered as intended in the real world, and ‘maintenance’ refers to how the program and/or benefits are sustained over time. Glasgow and colleagues [38] argue that “new information technologies may have greater reach, adoption, implementation and maintenance, and thereby greater public health impact” and thus the impact of this technology-based intervention may be greater than a simple efficacy-based evaluation would imply.

Intervention fidelity, which refers to whether an intervention is designed, delivered and received as intended, was evaluated using the five recommended domains of the National Institutes of Health (NIH) behaviour change consortium (study design, training providers, delivery of treatment, receipt of treatment and enactment of treatment skills) [28]. Detailed intervention fidelity reporting will inform recommendations on future enhancement strategies. ‘Study design’ relates to design choices made to ensure adequate testing of a hypothesis in relation to its underlying theory and clinical processes. ‘Training providers’ assesses whether providers have been satisfactorily trained and have the competencies required to deliver the intervention. ‘Delivery of treatment’ concerns whether the intervention is delivered as intended. ‘Receipt of treatment’ assesses whether patients understand and perform treatment-related behavioural skills and cognitive strategies during treatment delivery. ‘Enactment of treatment skills’ assesses whether the intervention influences patients’ performance of treatment-related behavioural skills and cognitive strategies in relevant real-life settings.

## Results

Thirty-five patients of the 71 (49%) potentially eligible patients consented to participate in this phase of the study. The average age of participants was 59 years and 54% were female. Further characteristics of participants can be found in Table 2. Results of the impact assessment are then presented, structured according to dimensions of the RE-AIM framework [22].

**Table 2:** Participant Characteristics

	Overall (*n* = 35)
Age (years), mean (SD)	59 (9)
Gender M, n (%)	16 (46)
BMI, median [IQR]	25.9 [23.7, 34.0]
Surgery Type*, n (%)	
Grade II	6 (17)
Grade III	29 (83)
CCI, mean (SD)	4.8 (1.9)
ARISCAT, n (%)	
High	7 (20)
Moderate	27 (77)
Low	1 (3)
Area of Residence, n (%)	
Capital or Metropolitan	11 (31)
Rural or Regional	24 (69)
State, n (%)	
VIC	30 (86)
TAS	4 (11)
NSW	1 (3)
Smoking status, n (%)	
Never smoked	17 (53)
Quit smoking longer than 8 weeks ago	13 (41)
Current smoker	2 (6)
Highest Level of education, n (%)	
Primary school	2 (6)
Secondary school	11 (34)
Trade school/TAFE	8 (25)
Undergraduate degree	7 (22)
Postgraduate degree	4 (13)

*Abbreviations*: *ARISCAT* Assess Respiratory Risk in Surgical Patients in Catalonia. Area of Residence determined by the Accessibility/Remoteness Index of Australia [39]. *Surgery Type determined using modified John Hopkins Surgical Category [30]

### Reach

**Fig. 3:**
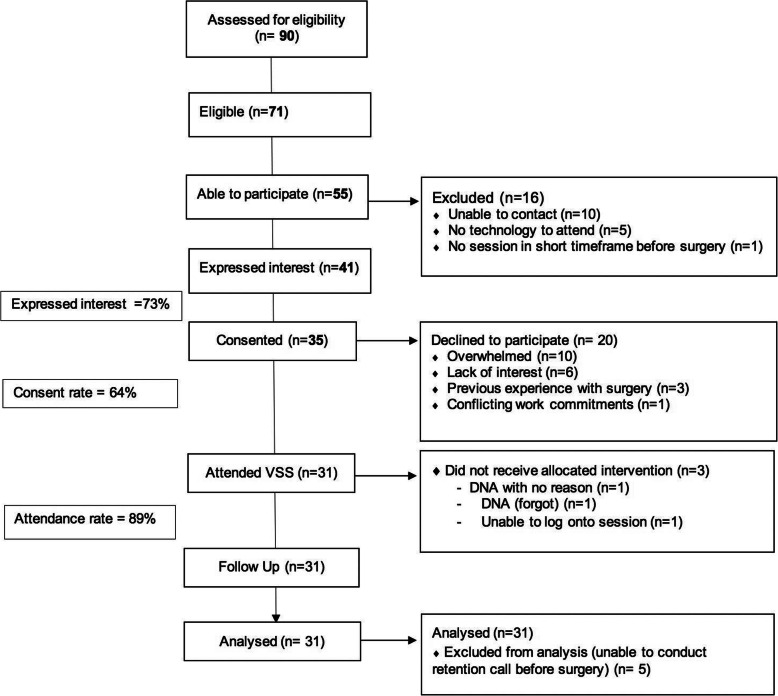
Study Flow

Over the four-month period from February to May 2020, 35 of the 71 eligible participants (49%) consented and 31 (89%) attended a webinar (Fig. 3). Twenty-four participants (69%) lived outside metropolitan areas with five (14%) participants from other Australian states.

Of those 20 participants who declined, reasons given were feeling ‘overwhelmed’ (50%), lack of interest (30%), previous surgery experience (15%) and unable to attend due to work commitments (5%). When discussing overwhelm as a barrier to participation many potential participants reported already being linked with other prehabilitation services prior to invitation to VSS. After reviewing medical records it became clear that, of those screened, 86 (96%) had already been seen by another member of the prehabilitation service, in which they may have already received some of the content covered in VSS.

An average of 1.5 attempts were needed to establish contact with a potential participant with an additional range of 0 to 5 attempts made from initial contact to participation or decline. Forty-one (73%) of screened potential participants expressed interest to participate in the program when contacted on the phone however six did not proceed to formally consent to the study when presented with the e-consent process. Three participants were unable to complete the e-consent process and instead completed hard-copy consent forms by mail.

Nine participants (29%) watched the session with a support person (family (89%) or friend (11%)) and thus carers were also reached with the intervention. Of participants who watched the session with family, examples of written feedback from the evaluation form included:

*“Excellent as I could have my family watch it with me and get a better understanding of what will happen direct from yourselves and not secondary from me”(Participant 15).*

*“This session has helped me feel more prepared for my surgery coming up... I will be watching the webinar with my family later today so they can help and support me” (Participant 33).*

### Efficacy

It is not possible at this stage to assess the clinical efficacy of VSS but for the purposes of evaluating impact, patient centred variables were used as proximal indicators of efficacy.

All participants who received the intervention completed the evaluation questionnaire and 26 participated in the follow up call prior to surgery. The remaining five were followed up after surgery due to short surgical timeframes prohibiting them being interviewed prior to surgery.

**Fig. 4:**
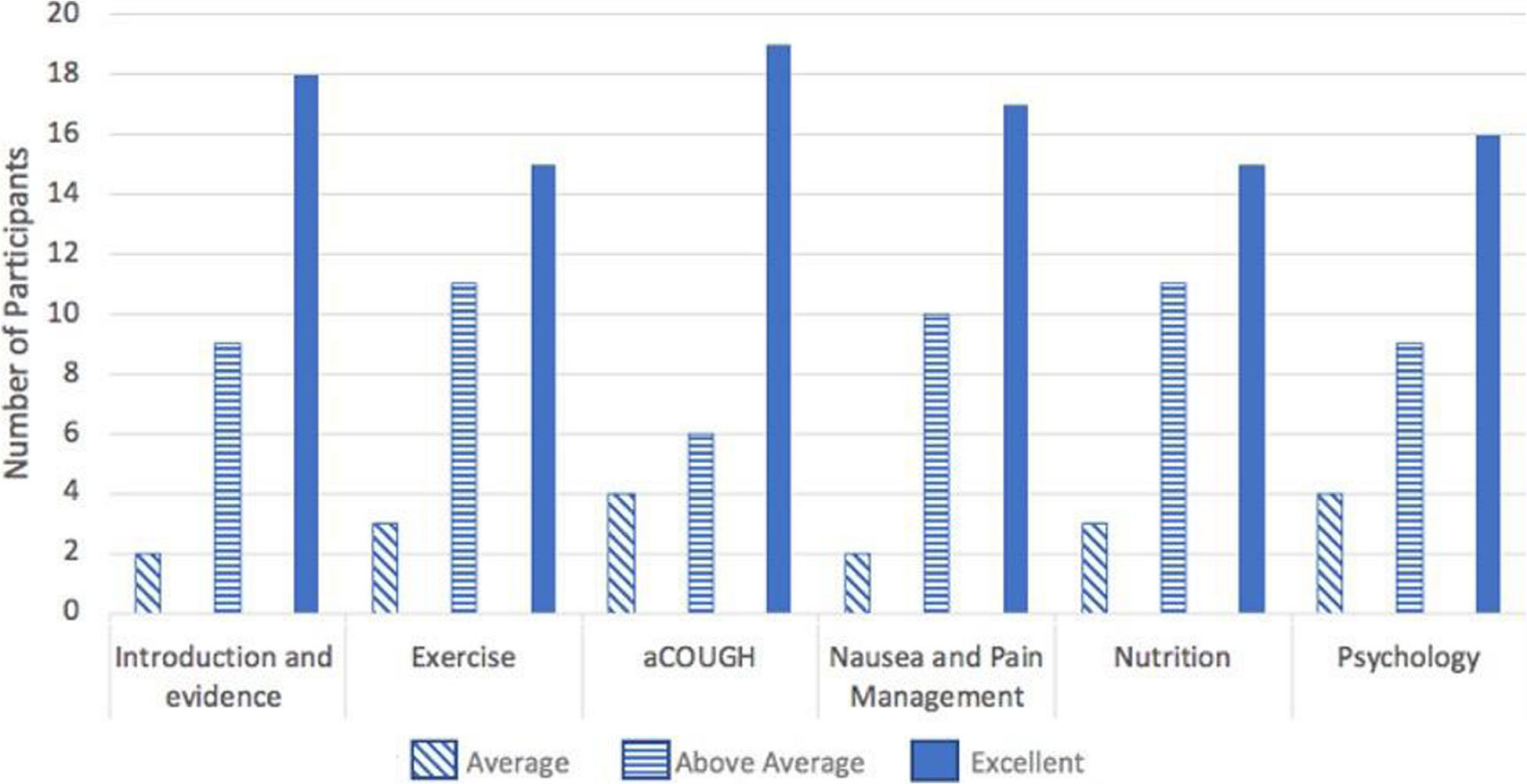
Individual Presentation Evaluations

The overwhelming majority (31; 97%) of participants who attended the online education session reported they would recommend it to others preparing for surgery. Participants’ level of agreement with satisfaction statements “I know what to expect after my surgery” (z = − 3.87, *p* = < 0.001) and “I am prepared for my experience after surgery” (z = − 3.21, *p* = 0.01) increased after the session. Participants’ agreement with the statement “I am prepared to follow instructions after surgery” was high at baseline and unchanged after the session (z = − 1.67, *p* = 0.10). On average individual presentations were rated above average or excellent by 26 (84%) of participants (Fig. 4).

Of participants who attended the webinar, 24 (77%) reported if given a choice they would attend the online education session as opposed to attending the hospital-based session.

*“I don't live near the hospital, so this was a great way to obtain all the information without a day of travel in heavy traffic” (Participant 5, evaluation form).*

*“It was brilliant. I didn’t have to travel, wait around or pay for parking”(Participant 25, interview).*

None of the participants reported a preference to watch the online session at a local community centre. Four participants (13%) reported they sought help from family/friends to set up the session however interestingly all participants reported that the system was easy to set up. All but one (97%) found it easy to use.

### Adoption

**Fig. 5:**
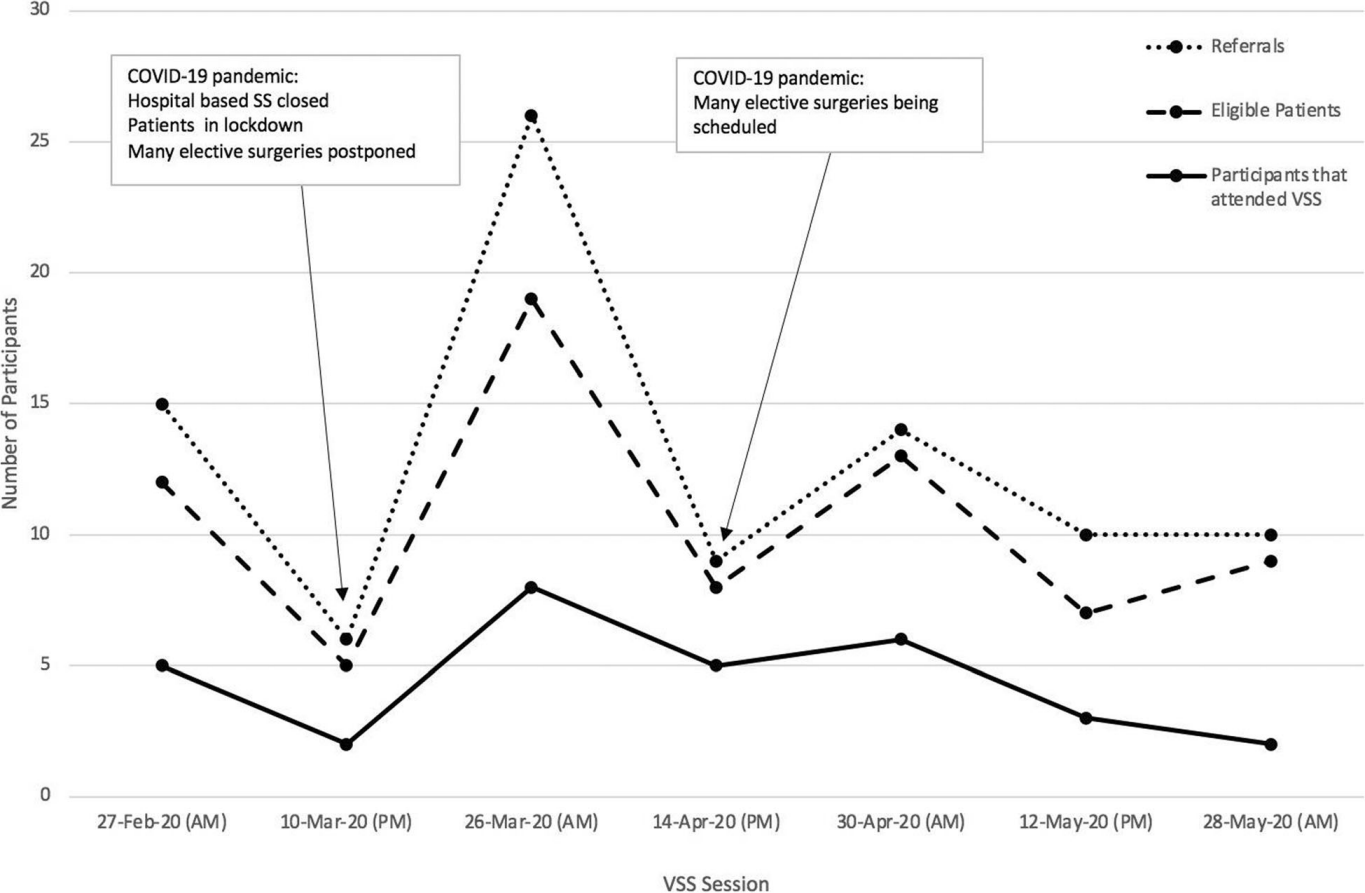
Referrals to each Virtual Surgery School session during Phase II

The intervention was introduced within a prehabilitation clinical service at a tertiary cancer hospital with an active perioperative medicine program and thus senior clinicians within the service were eager to promote VSS. Clinician referral (doctors, physiotherapists, nurses and dietitians) was the highest referral source (68% of total referred). Clinicians linked to the prehabilitation service were the highest referrers; however, referrals were received from specialist surgical nurses and consultant surgeons demonstrating a wider adoption of the online education session. Referrals also came from theatre waiting lists (19%), and multidisciplinary meeting lists (3%). Referral numbers fluctuated greatly with surgical cancellations due to the COVID-19 pandemic (Fig. 5).

### Implementation

Intervention fidelity was evaluated using the NIH framework [28] and thus is reported in the five recommended domains.

#### Fidelity of study design

Intervention components were pre-recorded and of similar duration (average 10 min) to ensure equivalent dose across conditions, the intervention was designed to last approximately 75 min. However it was becoming evident during implementation that many of the participants (96%) had already been seen by another member of the prehabilitation service and thus many participants may have received information similar to that delivered in the intervention webinar.

#### Fidelity of training for providers

The live facilitator underwent training as described in the methodology. In addition informal feedback was given on skill level and problem solving of technical issues, by members of the research team and lay volunteers, prior to implementation.

#### Fidelity of delivery of treatment

Intervention delivery lasted an average of 71 min (compared with 75 min as planned). Little variation was seen in the length of individual webinars, dependent on the number of questions asked by participants. All components of the session were delivered during the webinar using the standardised checklist as per the protocol.

#### Fidelity of receipt of treatment

Thirty-one participants attended the webinar. Four participants did not attend the online education session after consenting to participate and declined to attend a future session. Reasons for non-attendance were forgot to attend (1), unable to log into the technology and too overwhelmed with other things to try again (1). Two participants did not give a reason for non-attendance and were unable to be contacted. One participant left the session early after 44 min. This participant reported hearing this information recently from his treating team as the reason for leaving early. Participants asked only three questions across the seven online sessions throughout this pilot.

Almost all participants accessed the intervention from home with the exception of two participants. One prior to the COVID-19 lockdown, who did not have any technology at home, accessed the program from a local community centre with help of centre staff. Another accessed the intervention on his mobile phone whilst travelling to the hospital for treatment.

Seven (23%) participants reported watching one of the presentations again in the 2 weeks after the online education session. Participants reported aCOUGH was the most re-watched component with six views, followed by Psychology and Exercise with one view. Video repository data from the study period showed that videos of the online education session components were viewed 13 times with aCOUGH being the most viewed at four views followed by Psychology, Exercise and Pain with three, three and two views respectively. Videos were watched on average for 9 min and 12 s. It is unknown whether participants or family members viewed the videos in the repository.

Session information was recalled by all 26 participants who participated in the telephone interview before surgery. When prompted twenty-four (92%) remembered receiving information about breathing exercises and exercise more so than other components. Further recall and recognition data can be found in Table 3.

**Table 3:** Session Retention

Recall of specific information items from Virtual Surgery School (*n* = 26)		
Rationale for Surgery School Recalled	n	%
Improve general recovery	17	65.4
Return home faster	10	38.5
Patient empowerment	6	23.1
Prevent respiratory complications	2	7.7
Recall of most memorable content		
aCOUGH	13	50
Exercise	5	19.2
Nutrition	1	3.8
Psychology	2	7.7
General message/Intro	3	11.5
Other sections remembered		
Exercise	10	38.5
Nutrition	10	38.5
Pain management	7	26.9
Nothing	5	19.2
Psychology	4	15.4
aCOUGH	3	11.5
Intro	2	7.7
Use of Recommendations (*n* = 31)	n	%
Breathing Exercises		
Not at all	7	22.6
Once	1	3.2
Several times per week	5	16.1
Everyday	18	58.1
Mouthwash Y (%)	16	51.6
Visiting Dentist Y (%)	3	9.7
Maintained and increased exercise program, Y (%)	16	51.6

#### Fidelity of enactment of treatment skills

In the 2 weeks after the intervention over 50% of participants reported acting on exercise and oral care recommendations provided in the session, with the notable exception of attending the dentist which was very difficult at the time due to government COVID-19 precautions (Table 3).

## Discussion

The purpose of this paper was to evaluate the impact of an online education session using the RE-AIM framework and make recommendations to improve future impact and maintenance of the ongoing program. VSS was highly rated by participants, recommended to others preparing for surgery and resulted in a high level of enactment of recommendations in the 2 weeks after the session. VSS was successful in reaching a large proportion of people residing in rural and regional areas. Discussion below is reported according to the RE-AIM framework dimensions including an overall judgement (Low, Moderate or High) of impact (Additional file 3). The utility of using the RE-AIM framework for this study is also explored below.

### Reach

Ninety (77%) of patients having eligible surgeries across the pilot phase were screened for eligibility. Of those screened, 79% (71) met the study inclusion criteria with almost one in two (49%; 35/71) agreeing to attend the online education session. This exceeded rates reported for telehealth patient education sessions before total joint replacement [40]. However, despite the rapid promotion of telehealth technologies during the COVID-19 pandemic [41] and 89% of consenting participants receiving the intervention, we did not reach the anticipated recruitment of > 70% of all eligible patients. In a previous study at the same centre, 27% of patients preparing for major cancer surgery stated they would not participate in a prehabilitation program if telehealth/technology were used and 10% were unsure [42], therefore 70% may have been an unrealistic target for this patient group. Individual priorities in a stressful time, including during COVID 19, information already provided, personal commitments [43], issues with the consent process as well as technology skills needed by participants were possible reasons for this difference and should be carefully considered during this period.

**Fig. 6:**
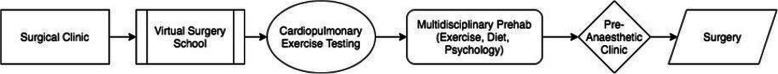
Proposed New Perioperative Prehabilitation Pathway

Ninety-six percent of screened participants had already been seen by a member of the prehabilitation service prior to VSS. Although clinical champions are generally a source for improving participation [42], especially when they endorse a program, it was noted that many patients declining due to ‘overwhelm’ (50%) felt they had enough information already prior to the invitation to VSS. Streamlining of services may be required to provide people preparing for cancer surgery key information, utilise available resources and reduce burden to patients while improving health service efficiencies. Streamlining would also avoid possible contamination of evaluation results across interventions. As VSS was a general intervention and not tailored to patient-specific comorbidities and fitness, we recommend that attendance at VSS be brought forward in the prehabilitation pathway to address these issues (Fig. 6).

This study used an electronic consent process and found that six participants who originally expressed interest did not proceed to signing the electronic consent form and thus participate. It is unclear from this study whether this affected reach of the intervention. It is thus essential to consider resource planning and recruitment methods independently when assessing the effect of complex interventions [44]. A moderate level of impact on reach was achieved.

### Efficacy

Overwhelmingly participants would recommend VSS to others preparing for surgery and rated the session components highly, similar to the in-hospital session on which it was based [21]. Patients showed an improvement in their self-reported preparedness for surgery and experience post-operatively. Pre-operative patient education that resulted in patients feeling more prepared and may have decreased the rate of post-operative complications. This outcome is reported is several other studies [21, 45]. A high level of impact on efficacy was achieved.

### Adoption

Adopters of the program were mainly from the prehabilitation clinical service. However referrals from specialist nurses and surgeons demonstrated a wider adoption outside of the service. This maybe further enhanced with the use of audit-feedback loops [46] to enable referring clinicians to visualise the effects of VSS and thus further support referral. A moderate level of impact on adoption was achieved.

### Implementation

The implemented session was of similar length to previously described preoperative education sessions rated as appropriate by patients [21] with high retention, similar to other pre-operative education sessions [34], and enactment of recommendations. This is possibly due to the delivery setting and possibly influenced by the high number of participants being seen by specialist prehabilitation services alongside VSS that may have further reinforced recommendations, suggested in a recent study of cancer patients preparing for major surgery as a motivating factor [43]. A moderate-high impact on implementation was achieved.

#### Lessons learnt and recommendations

Based on the RE-AIM model several recommendations are offered for providers who wish to implement a similar program. Recommendations will also inform future maintenance of this program. It is recommended that *Reach* be improved by (1): removing electronic consent barriers (2); providing technology for those who do not have it; and (3) streamlining pre-operative services that may target the same potential participants to ensure patients do not feel overwhelmed by commitments and receive the same information multiple times.

To enhance *Adoption* (4) it is recommended that researchers consider further promotion of VSS to likely referring staff and potential participants. Widely circulated results of VSS on outcomes including patient satisfaction and clinical outcomes with the use of feedback loops is also recommended to further facilitate referrals to the program.

*Implementation* could be improved by *Receipt of treatment* could be further evaluated by (5) changing the order in which information is presented to evaluate how that changes retention of information by participants (6); the inclusion of interactive questions and/or a post-session knowledge quiz for participants and the possibility of live presenters could be considered. Lastly (7) *Enactment of treatment skills* could be further evaluated by objective tracking of participant behaviour in the pre-operative period, e.g. pedometers.

#### Using RE-AIM

The RE-AIM framework served as an important tool for evaluating the impact and future impact of VSS. Each RE-AIM dimension was used to identify key areas for improvement for future maintenance. However the short duration of the study phase did not allow for the assessment of the maintenance dimension of the RE-AIM framework, which Glasgow and colleagues [47] recommended should be at least 2 years. Long term effectiveness and program sustainability were unable to be established. Issues with maintenance have been reported in other studies [48] and program developers should consider and plan for the timeframe required to successfully evaluate newly implemented programs.

### Strengths and limitations

This paper benefits from multiple methods of data collection, thorough description of the intervention and is strengthened by application of a framework on which to structure the investigation. It is also supported by senior medical clinician recommendation, a referral source that has been reported to affect participation within this patient group [42]. However limitations of this study include the reliance on self-report and questionnaires to assess efficacy and fidelity, which had not previously been validated. This study may also be limited by the inability to deliver ongoing promotion to facilitate within the hospital due to the restrictions and priorities during the COVID-19 pandemic, which likely affected reach of the program. A potential limitation of this study is that there was only one trained facilitator. Whilst this was not an issue in this study, we recommend having at least two trained facilitators for succession planning and sustainability of the intervention. Nonetheless, this study provides recommendations for developers and providers seeking to implement future telehealth interventions.

## Conclusion

Telehealth alternatives to hospital based pre-operative education are well received by patients preparing for major cancer surgery. However careful evaluation of implementation strategies alongside clinical and cost effectiveness in future studies is essential to support addition of this intervention into standard care.

## Supplementary Information

**Additional file 1.** TIDieR checklist item descriptions.

**Additional file 2.** Study questionnaires and retention phone call script and scoring template.

**Additional file 3.** RE-AIM impact scoring.

## Data Availability

Data is not available as it is part of an ongoing research study. After completion of the ongoing study, data will be provided by the PI upon request.
