# Supplementary material for: Implementing a telehealth prehabilitation education session for patients preparing for major cancer surgery
Source: BMC Health Serv Res. 2021 May 10;21:443. doi: 10.1186/s12913-021-06437-w (PMC8108411; doi:10.1186/s12913-021-06437-w)
Supplement: Supplementary file 1 — Additional file 1. TIDieR checklist item descriptions. [file 12913_2021_6437_MOESM1_ESM.docx]

**Additional File 1**

TIDieR checklist item descriptions taken from Hoffman TC, Glasziou PP, Boutron I, Milne R, Perera R, Moher D, Altman DG, Barbour V, Macdonald H, Johnston M, Lamb SE, Dixon-Woods M, McCulloch P, Wyatt JC, Chan A-W, Michie S (2014) Better reporting of interventions: template for intervention description and replication (TIDieR) checklist and guide. BMJ : British Medical Journal 348:g1687. doi:10.1136/bmj.g1687

| **Item Number** | Description |
| --- | --- |
| **Item 1.** | Brief name |
| **Item 2.** | Why: Describe any rationale, theory, or goal of the elements essential to the intervention. |
| **Item 3.** | What (materials): describe any physical or informational materials used in the intervention including those provided to participants or used in intervention delivery or in training of intervention providers |
| **Item 4.** | What (procedures): describe each of the procedures, activities, and/or processes used in the intervention, including any enabling or support activities |
| **Item 5.** | Who Provided: for each category of intervention provider (for example, psychologist, nursing assistant), describe their expertise, background and any specific training given. |
| **Item 6.** | How: Describe the modes of delivery (such as face to face or by some other mechanism, such as internet or telephone) of the intervention and whether it was provided individually or in a group. |
| **Item 7.** | Where: Describe the type(s) of location(s) where the intervention occurred including any necessary infrastructure or relevant features. |
| **Item 8.** | When and how much: Describe the number of times the intervention was delivered and over what period of time including the number of sessions, their schedule and their duration, intensity or dose. |
| **Item 9.** | Tailoring: If the intervention was planned to be personalised, titrated or adapted, then describe what, why, when and how. |
| **Item 10.** | Modifications. |
| **Item 11.** | How well (planned): if intervention adherence or fidelity was assessed, describe how and by whom, and if any strategies were used to maintain or improve fidelity, describe them. |
| **Item 12.** | How well (actual): if intervention adherence or fidelity was assessed, describe the extent to which the programme was delivered as planned |
