# Supplementary material for: Implementing a telehealth prehabilitation education session for patients preparing for major cancer surgery
Source: BMC Health Serv Res. 2021 May 10;21:443. doi: 10.1186/s12913-021-06437-w (PMC8108411; doi:10.1186/s12913-021-06437-w)
Supplement: Supplementary file 2 — Additional file 2. Study questionnaires and retention phone call script and scoring template. [file 12913_2021_6437_MOESM2_ESM.docx]

**Additional File 2**

**Pre-session Questionnaire**

| **Please indicate how strongly you disagree/agree with statement*** | Strongly Disagree | Disagree | | Neutral | Agree | Strongly agree |
| --- | --- | --- | --- | --- | --- | --- |
| I know what to expect after surgery | □ | □ | | □ | □ | □ |
| I’m prepared for experience post-surgery | □ | □ | | □ | □ | □ |
| I am prepared to follow instructions such as prehabilitation/rehabilitation | □ | □ | | □ | □ | □ |
| What is your highest level of education? | | | □ Completed primary school  □ Completed secondary school  □ Completed trade school/TAFE  □ Completed undergraduate degree  □ Completed postgraduate degree | | | |
| Do you currently smoke? | | | □ Yes  □ I’ve never smoked  □ I quit smoking | | | |
| If Y, how long have you smoked for? How many do you smoke on an average day? | | | ____________ | | | |
| If Y, how long ago did you quit? | | | ______________ | | | |
| If Y, how many did you smoke on an average day? | | | ______________ | | | |

**Post-session Evaluation Questionnaire**

| **Please indicate how strongly you disagree/agree with statement*** | Strongly Disagree | Disagree | Neither agree or disagree | Agree | Strongly agree |
| --- | --- | --- | --- | --- | --- |
| I know what to expect after surgery | □ | □ | □ | □ | □ |
| I’m prepared for experience post-surgery | □ | □ | □ | □ | □ |
| I am prepared to follow instructions such as prehabilitation/rehabilitation | □ | □ | □ | □ | □ |
| **Please rate how helpful you found the following sessions.** | **Poor** | **Below Average** | **Average** | **Above Average** | **Excellent** |
| Introduction/Why do I need Surgery School | □ | □ | □ | □ | □ |
| Exercise Prehabilitation | □ | □ | □ | □ | □ |
| aCOUGH | □ | □ | □ | □ | □ |
| Nausea and pain management | □ | □ | □ | □ | □ |
| Nutrition | □ | □ | □ | □ | □ |
| Psychology | □ | □ | □ | □ | □ |
| Would you recommend this session to others preparing for surgery? | □ No  □ Yes | | | | |
| Where would you have preferred to attend Surgery School? | □ Hospital based Surgery School  □ Virtual Surgery School at home  □ Virtual Surgery School at local hospital/health centre | | | | |
| Was the technology for Surgery School easy to set up? | □ No  □ Yes | | | | |
| Was the technology for Surgery School easy to use? | □ No  □ Yes | | | | |
| Did you need help to set up the webinar? | □ No  □ Yes | | | | |
| Do you have any other comments that might be useful about your experience using Virtual Surgery School? | ____________________________________ | | | | |

**Retention Phone Call Script and Scoring Template**

**Intro:** Hi, I’m calling from the Department of Anaesthesia at Peter Mac and I’d like to ask you some questions about how you feel about the education you were given two weeks ago.

I know that two weeks ago you attended an education session called Surgery School via a live webinar where you were given lots of information about how to prepare for surgery.

S**ince attending the Surgery School, have you re-watched any of the Surgery School videos?**

| Yes | No |
| --- | --- |

**If Y, which videos did you watch again?**

| Videos | If selected: how many times |
| --- | --- |
| Introduction to Surgery School |  |
| Exercise Prehabilitation |  |
| aCOUGH (breathing exercises) |  |
| Nausea and pain management |  |
| Nutrition |  |
| Psychology |  |

1. **What pieces of information you received that day really stand out in your memory?**

| *Please circle the first mentioned:* | | | | | | |
| --- | --- | --- | --- | --- | --- | --- |
| aCOUGH | Exercise | Nutrition | Pain management | ERAS | Psychology | Other |

| *Please circle all others mentioned:* | | | | | | |
| --- | --- | --- | --- | --- | --- | --- |
| aCOUGH | Exercise | Nutrition | Pain management | ERAS | Psychology | Other |

1. **Do you remember learning about breathing exercise exercises during the webinar?**

| Yes | No |
| --- | --- |

1. **What do you remember about the breathing exercises that were described? Circle as many as appropriate for this participant**

| Nothing | Research | Coughing | Early ambulation | Lung physiology or mucociliary clearance |
| --- | --- | --- | --- | --- |
| Circulation exercises | Preventing pneumona | Preoperative fitness optimisation | Other: _______________ | |

1. **Can you tell me what the 4 phases of the breathing exercises are? (Circle all described by participant)**

| Breathing control or relaxed breathing | Deep breaths | Huff | Cough |
| --- | --- | --- | --- |

1. **Tell me why these things are important.**

| Prevent respiratory/lung complications | Mucociliary clearance | Lung recovery | Improve General recovery | Heart/circulation |
| --- | --- | --- | --- | --- |
| Other: | | | | |

1. **How often have you practiced these breathing exercises since listening to the webinar?**

| Not at all | Once | Several times per week | Everyday |
| --- | --- | --- | --- |

1. **Can you tell me what aCOUGH stands for? (please circle all participant could recall)**

| Active cycle of breathing technique | Cough (or deep breath and cough) | Oral care | Understanding (or understanding aCOUGH) | Head of the bed elevated (or head of the bed up) |
| --- | --- | --- | --- | --- |
| Other: | | | | |

1. **Since attending the webinar have you made an appointment or attended you dentist/dental hygienist?**

| No | Yes | Other: ___________ |
| --- | --- | --- |

1. **Since attending the webinar have you started using a mouthwash?**

| No | Yes | Other: ___________ |
| --- | --- | --- |

1. **Do you remember learning about exercise during that session?**

| No | Yes | Other: ___________ |
| --- | --- | --- |

1. **What do you remember about the exercise during that session?**

| Improve fitness before surgery | Research | Frequency/Duration/Type | Other: __________________ |
| --- | --- | --- | --- |
| Anaerobic Exercise | Aerobic Exercise | Prevent complications/Problems |  |

1. **Can you tell me how much exercise you should be doing before the operation per week?**

| 150mins moderate exercise or 75 mins vigorous exercise | 2-3 resistance sessions per week | Other: ___________ |
| --- | --- | --- |

1. **Since attending the webinar have you started or increased your exercise program?**

| No | Yes | Other: ___________ |
| --- | --- | --- |

1. **Do you remember learning about pain relief during that session?**

| Yes | No |
| --- | --- |

1. **What do you remember about the pain relief during that session?**

| Types of pain relief (tablets/PCA etc) | Ensuring pain well managed | Ensuring pain is manageable to do deep breaths, cough and walk around | Reasons for post-operative pain: |  |
| --- | --- | --- | --- | --- |
| Other: __________________________ | | | | |

1. **Do you remember learning about what to eat during the session?**

| Yes | No |
| --- | --- |

1. **What do you remember about what to eat to prepare for surgery?**

| Increased need for protein and energy before surgery | Muscle loss | Increase muscle before surgery | Helps improve functional recovery | Eat and drink soon after surgery |
| --- | --- | --- | --- | --- |
| Poor nutrition = impaired healing | Complications | Poor nutrition is linked with poorer surgical outcomes | Weight maintenance | Other: __________________________ |

1. **You learnt about a lot of things during the webinar. Can you tell me why these things are important?**

| Prevent respiratory/lung complications | Improve general recovery | Return home faster | Other: ______________________ |
| --- | --- | --- | --- |

1. **Do you think the way this information was presented helped you remember it?**

1. **Why do you think that?**

___________________________________________________________________________
