# Supplementary material for: Implementing a telehealth prehabilitation education session for patients preparing for major cancer surgery
Source: BMC Health Serv Res. 2021 May 10;21:443. doi: 10.1186/s12913-021-06437-w (PMC8108411; doi:10.1186/s12913-021-06437-w)
Supplement: Supplementary file 3 — Additional file 3. RE-AIM impact scoring. [file 12913_2021_6437_MOESM3_ESM.docx]

**RE-AIM Impact Scoring (Low, Moderate, High)**

| **Dimension** | **Aim** | | **Decision** | | **Rationale** |
| --- | --- | --- | --- | --- | --- |
| **Reach** | Participation and representativeness of the target population.  Target population: Patients preparing for major abdominal cancer surgery who were unable to, or did not wish to, attend the hospital based session. | | Moderate | | 31/117 total major cancer surgeries attended however ‘true’ denominator of patients who did not attend the hospital session and met the inclusion criteria isn’t know (data on surgeries not screened unknown) It is likely to be >25%. |
| **Efficacy** | Whether the program influenced the target outcomes. | | High | | High level of patient variables – satisfaction/recommendation/improvement.   - 31/97% would recommend it to others - Improvement in satisfaction p<0.001 - Improvement in preparedness p=0.01 - Individual presentation were rated above average or excellent by 26 (84%) of participants - 24 (77%) reported if given a choice they would attend the online education session as opposed to attending the hospital-based session. |
| **Adoption** | Uptake by agencies and settings. | | Moderate | | Downgraded one as 68% were referred by clinicians from the rehab service. |
| **Implementation** | | | | | |
| *Fidelity of study design* | | Design choices made to ensure adequate testing of a hypothesis in relation to its underlying theory and clinical processes. | High | - Same dose given within conditions (one session) - Ensure equivalent dose across conditions (each presentation was similar length) - Further planning needed for implementation sets (treatment provider drop-out) however was not an issue in this study but forms a recommendation going forward. | |
| *Fidelity of training for providers* | | Whether providers have been satisfactorily trained and have the competencies required to deliver the intervention. | Moderate | - Non formalised training process with members of the research team and lay volunteers providing informal feedback. | |
| *Fidelity of delivery of treatment* | | Whether the intervention of delivered as intended. | High | - 100% checklist completion (relying on self-report which is subject to bias and ideally other forms of delivery assessment would be ideal in future studies). | |
| *Fidelity of treatment receipt* | | Whether the patients understand and perform treatment-related behavioural skills and cognitive strategies during treatment delivery. | High | - Large proportion (97%) of participants attended the whole session (only one left early) - Session information recalled by all 26 participants interviewed before surgery. | |
| *Fidelity of enactment of treatment skills* | | Whether the intervention influences patients’ performance of treatment-related behavioural skills and cognitive strategies in relevant real-life settings. | Moderate | - Over 50% of participants reported acting on exercise and oral recommendations provided in the session. - 24 (77%) practised the breathing exercises with 58% practising them every day. | |
| **Overall** | |  | Moderate-High |  | |
